# Targeted Isolation of Antioxidant Constituents from *Plantago asiatica* L. and In Vitro Activity Assay

**DOI:** 10.3390/molecules25081825

**Published:** 2020-04-16

**Authors:** Yuanyang Dong, Qihang Hou, Meng Sun, Jingjing Sun, Bingkun Zhang

**Affiliations:** State Key Laboratory of Animal Nutrition, Department of Animal Nutrition & Feed Science, College of Animal Science & Technology, China Agricultural University, Beijing 100193, China; yuanyangdong@cau.edu.cn (Y.D.); qihanghou1992@163.com (Q.H.); sunmengdk@163.com (M.S.); jingjingsun0816@126.com (J.S.)

**Keywords:** antioxidants, compound isolation, DPPH, *Plantago asiatica* L., aesculetin, apigenin

## Abstract

*Plantago asiatica* L. is widely distributed in Eastern Asia and a commonly used drug in China, Korea, and Japan for diuretic and antiphlogistic purposes. In this experiment, the present study was performed to isolate antioxidant molecules based on the DPPH scavenging activity assay and discover the bioactive compounds which contributed to performing the function of *Plantago asiatica* L. Each faction was chosen for further isolation guided by DPPH scavenging activity assay. Afterwards, two potential bioactive molecules, aesculetin and apigenin, were isolated for in vitro antioxidant activity in cells. Hydrogen-peroxide-induced oxidative stress led to decreased cell viability, impaired intercellular junction, and damage to the cell membrane and DNA. Furthermore, aesculetin ameliorated decreased cell viability induced by hydrogen peroxide via upregulation of antioxidant related genes, and apigenin also protected against H_2_O_2_ mainly by improving the glutathione (GSH) antioxidant system, such as increasing the activity of glutathione peroxidase (GPX), glutathione reductase (GR), and the ration of GSH/glutathione disulfide (GSSG). Above all, these findings suggest that aesculetin and apigenin may be bioactive compounds for antioxidant function in *Plantago asiatica* L.

## 1. Introduction

Oxidative stress is a consequence of an increased generation of free radicals and reduced antioxidant defense against free radicals [1]. Oxidative stress could result in DNA damage, and oxidative DNA adducts such as 8-oxodG have been involved in the tumorigenic process [2]. Antioxidant supplementation has become an increasingly popular practice to maintain body function [3]. However, several synthetic antioxidants such as butylated hydroxyanisole (BHA), butylated hydroxytoluene (BHT), and tert-Butylhydroquinone (TBHQ) have been reported to be harmful at high levels in animal experiments, and TBHQ is restricted in some countries, such as Canada and Japan [4]. TPlants are the richest sources of natural antioxidants. However, phytochemicals such as flavonoids are not easily absorbed, and higher concentration of flavonoids is more likely to be achieved in the lumen of the gastrointestinal tract where the flavonoids exert their antioxidant function [5]. The interaction between endogenous reactive oxygen species (ROS) and dietary antioxidants firstly takes place in the gastrointestinal tract [6]. There are several important aspects that should be carefully considered when it comes to the application of antioxidants, including their scavenging capacities, possible role in the endogenous antioxidant network, and their bio-viability [7].

*Plantago asiatica* L. is widely distributed in Eastern Asia and a commonly used drug in China, Korea, and Japan for diuretic and antiphlogistic purposes [8]. Phytochemical studies have shown that the *Plantago* genus contains a great number of natural products such as iridoids, flavonoids, tannins, triterpennes, saponins, and sterols [9]. Crude extracts of Asiatic plantain were found to significantly decrease DNA damage in lymphocytes caused by high-fat meals [10], but the effective molecules in the *plantago* were not identified. This study was designed to discover the bioactive molecules which contributed to performing the function of *Plantago asiatica* L. and exploring the possible mechanism.

## 2. Results and Discussion

### 2.1. Targeted Isolation of Antioxidant Constituents from Plantago asiatica L. Based on DPPH Scanvenging Assay

Seven extracts were obtained from superfine power of *Plantago Asiatica* L. Petroleum ether (15.61 g), dichloromethane (12.53 g), ethyl acetate (3.44 g) and ethyl acetate:methanol (10:1, *v*/*v*) gradient elution (148.34 g), ethyl acetate:methanol (5:1, *v*/*v*) (77.0 g), ethyl acetate:methanol (2:1, *v*/*v*) (30.25 g), and methanol (105.12 g) extracts were obtained through solid–liquid extraction. These solvents were chosen because they cover a wide range of polarities, allowing the fractionation of the substances. The different extract weights could be due to the characteristics and contents of the chemicals in the extracts. Additionally, ethyl acetate:methanol (10:1) produced the highest extraction yield of *Plantago asiatica* L., which suggested that most metabolites were moderately polar.

DPPH assay is one of the most commonly used methods due to its efficiency and simplicity. The free radical scavenging capacity of a sample is expressed as efficient concentration (EC_50_), which is useful for comparing results due to its independence of the sample concentration [11]. The antioxidant activity of different extracts and fractions on DPPH radicals was tested, and their corresponding EC_50_ values are shown in Table 1. Ethyl acetate:methanol gradient elution showed the highest radical inhibitory activity with EC_50_ 0.086–0.117 mg/mL, followed by ethyl acetate extract, methanol extract, and dichloromethane extract. In addition, as ethyl acetate:methanol (10:1) gave the highest yield, acetate:methanol (10:1) extract was chosen for further chromatographic separation, and six fractions, namely, F1–F6, were obtained based on the images on thin layer chromatography. Among the six fractions, F4 and F6 displayed the lowest EC_50_ values for the DPPH radical quench assay. Aesculetin (P1) was isolated from F4 via recrystallization.

**Figure 1:**
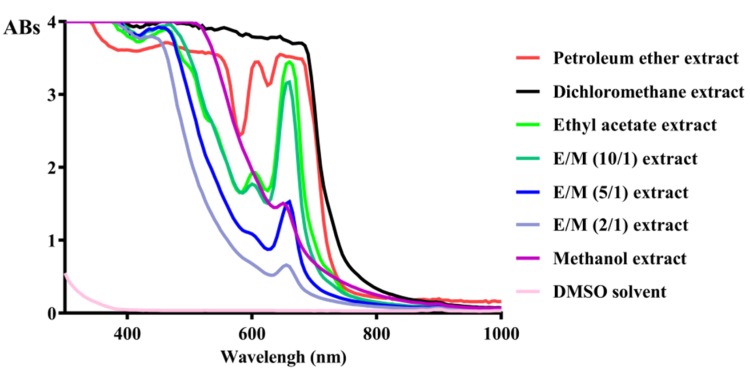
Wavelength scanning of different extracts.

The absorbance of different extracts between 0 and 1000 nm wavelength was scanned (Figure 1). Ethyl acetate and ethyl:methanol (10:1) extract showed a similar shape of absorbance pattern. Similarly, in another study aimed at characterizing the different polarity extracts obtained from *Plantago major*, methanol and ethyl acetate extracts had higher phenol concentration than dichloromethane and hexane, and only ethyl acetate had highest flavonoid concentration, including gallic acid, luteolin, quercetin, catechin, and galangin [12]. Thus, ethyl acetate extract was further analyzed. Apigenin (P2) was isolated from ethyl acetate extract.

### 2.2. Structural Determination of the Isolated Compounds

Four compounds were successfully isolated from the most active fractions of the *Plantago Asiatica* L. P1 was obtained as yellow needle-like crystals; mp. 268–270 °C; ESI-MS *m*/*z*: 179 [M + H]^+^; ^1^H-NMR (500 MHz, DMSO-*d*_6_) δ: 7.85 (1H, d, *J* = 9.5 Hz, H-4), 6.15 (1H, d, *J* = 9.5 Hz, H-3), 6.97 (1H, s, H-5), 6.74 (1H, s, H-8), 10.20 (1H, s, OH), 9.39 (1H, s, OH). According to the published literature, the above data were consistent with the report, and compound P1 was identified to be aesculetin [13]. P2 was isolated as a faint yellow powdered crystal, mp. 347–348 °C. ESI-MS *m*/*z*: 271 [M + H]^+^; ^1^H-NMR (500 MHz, DMSO-*d*_6_) δ:12.96 (1H, s, 5-OH), 7.92 (2H, d, *J* = 9.0 Hz, H-2′, 6′), 6.92 (2H, d, *J* = 9.0 Hz, H-3′, 5′), 6.78 (1H, s, H-3), 6.48 (1H, d, *J* = 2.0 Hz, H-8), 6.19 (1H, d, *J* = 2.0 Hz, H-6). From these observations and through comparison with literature NMR data [14], we concluded that P2 was apigenin.

### 2.3. Hydrogen-Peroxide-Induced Oxidative Stress in Caco-2 Cells

**Figure 2:**
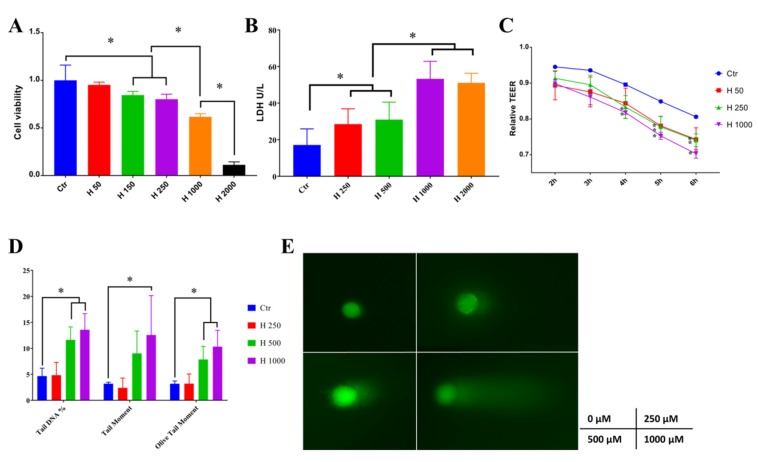
Establishment of a hydrogen peroxide-induced oxidative stress Caco-2 cell model. (**A**) Hydrogen peroxide significantly decreased cell viability. (**B**) Hydrogen peroxide above 250 μM significantly increased the lactate dehydrogenase (LDH) leakage from the cell. (**C**) Transepithelial electrical resistance (TEER) of Caco-2 was significantly decreased by hydrogen peroxide. (**D**,**E**) Tail DNA, tail moment, and olive tail moment was significantly unregulated by hydrogen peroxide, which were measured by comet assay (^*^ *p* < 0.05).

Hydrogen peroxide (H_2_O_2_) above 150 μM significantly decreased cell viability, and 1000 μM H_2_O_2_ further reduced cell viability to about 50% (Figure 2A). Similar results that 1 mM H_2_O_2_ treated for 6 h significantly reduced the cell viability of Caco-2 were observed [15]. Additionally, treatments with 0.8 mM H_2_O_2_ for 24 h led to increased release of lactate dehydrogenase (LDH), an indicator of cell membrane injury in IPEC-J2 cells [16]. LDH is a stable intracellular enzyme which can be released into the cell culture medium upon damage of the plasma membrane [17]. The leakage of LDH under treatment of H_2_O_2_ was measured in this study (Figure 2B). A total of 250–2000 μM H_2_O_2_ significantly increased the LDH level in the culture media, which indicated that H_2_O_2_ can lead to cell membrane damage. Transepithelial electrical resistance (TEER) was regarded as an indicator of monolayer integrity and paracellular permeability [18]. In Figure 2C, 250–1000 μM H_2_O_2_ decreased the TEER after 4 h treatment of hydrogen peroxide. Consistent with our experiments, a previous study showed that treatment of 500 μM H_2_O_2_ for 6 h also caused a significant decrease in TEER in Caco-2 cell monolayers [19]. ROS can be tumorigenic by inducing DNA damage, leading to a genetic lesion that initiates tumorigenicity [20]. Moreover, as shown in Figure 2D, 1000 μM H_2_O_2_ significantly upregulated the tail DNA percentage, tail moment, and olive tail moment, which were measured by comet assay, as shown in Figure 2E. Similarly, in another study, DNA damage as measured by comet assay was significantly high at concentration >100 μM H_2_O_2_ compared to that of the control in Caco-2 cells [21]. Above all, H_2_O_2_ treatment led to decreased cell viability, which may result from impaired cell membranes, damaged intercellular junctions, and DNA damage. Concentration of 1000 μM H_2_O_2_ was selected for the following study to induce cell oxidative stress.

### 2.4. Aesculetin and Apigenin Ameliorated Oxidative Damages Induced by Hydrogen Peroxide through Different Mechanisms

**Figure 3:**
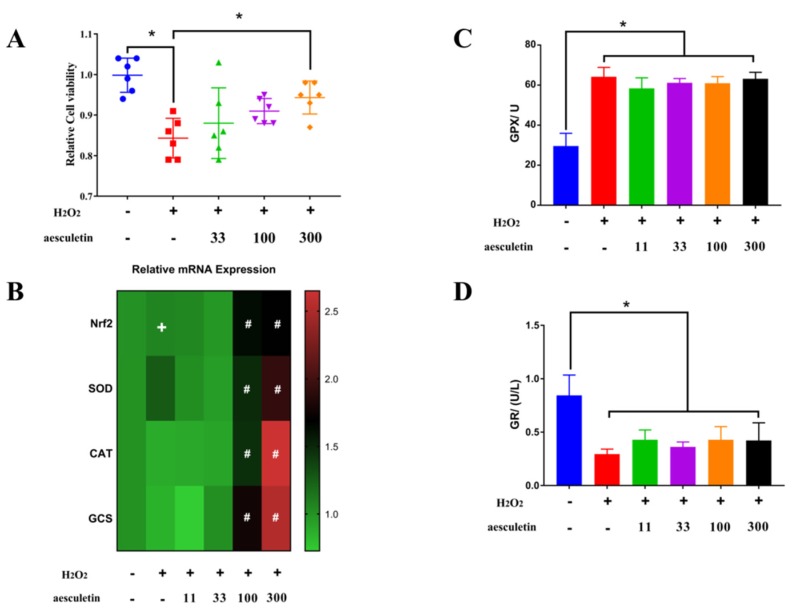
Aesculetin ameliorated decreased cell viability induced by hydrogen peroxide via upregulation of antioxidant related genes. (**A**) Aesculetin significantly ameliorated the decreased cell viability caused by hydrogen peroxide. (**B**) H_2_O_2_ slightly upregulated the mRNA expression of Nrf2 but 100 and 300 μg/mL aesculetin dramatically increased the gene expressions of Nrf2, superoxide dismutase (SOD), catalase (CAT), and glutamyl cysteine synthetase (GCS) compared to H_2_O_2_ treatment. (**C**,**D**) H_2_O_2_ treatment significantly improved the activity of glutathione peroxidase but suppressed the activity of glutathione reductase. (^*^ *p* < 0.05, ^+^ *p* < 0.05 vs. control, ^#^ *p* < 0.05 vs. H_2_O_2_ treatment).

Aesculetin significantly ameliorated the decreased cell viability caused by hydrogen peroxide (Figure 3A). Nuclear factor-E2–related factor 2 (Nrf2) is a transcription factor that is sensitive to oxidative stress and promotes the transcription of a wide variety of antioxidant genes, such as superoxide dismutase (SOD), catalase (CAT), glutathione S-transferase (GST), heme oxygenase (HO-1), gamma-glutamine cysteine synthase (γ-GCS), and glutathione peroxidase (GPX) [22]. In our study, aesculetin at 100 and 300 μg/mL dramatically enhanced the mRNA expression of Nrf2, and its downstream genes SOD, CAT, and GCS compared to H_2_O_2_ treatment, while H_2_O_2_ treatment only slightly increased the mRNA of Nrf2 (Figure 3B). Several kinds of natural and synthetic compounds were reported to activate the Nrf2/Keap1/ARE system: (1) diphenols, quinones, and phenylenediamines; (2) natural components from plants, such as curcumin, resveratrol, luteolin, and quercetin; (3) hydrogen peroxide, 4-*tert*-butyl hydrogen peroxide; and (4) components rich in trace elements such as selenium, arsenic, and other substances [23]. Therefore, aesculetin with an *ortho*-hydroxyl structure can further activate Nrf2 and enhance the transcription of SOD, CAT, and GPX, which was observed in this experiment. H_2_O_2_ significantly improved the activity of glutathione peroxidase but inhibited the activity of glutathione reductase (GR) (Figure 3C,D). However, aesculetin did not show any significant effect on the activity of GPX and GR.

**Figure 4:**
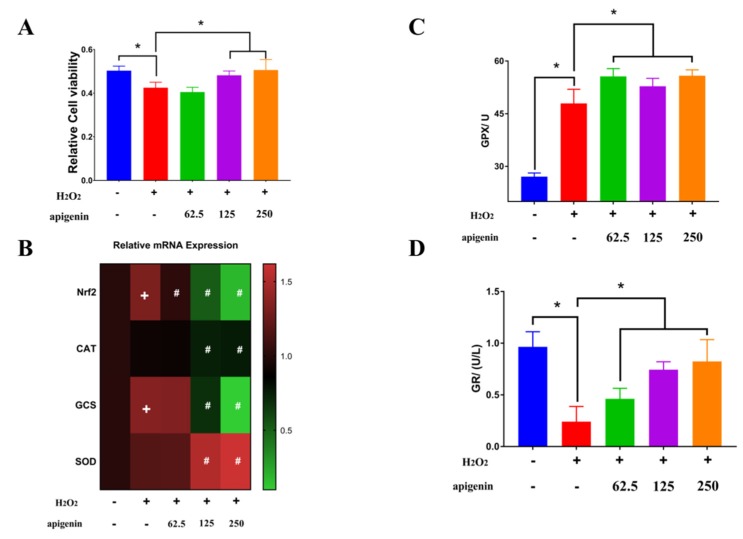
Apigenin ameliorated decreased cell viability induced by hydrogen peroxide through enhancement of glutathione peroxidase (GPX) and glutathione reductase (GR). (**A**) Apigenin ameliorated decreased cell viability induced by hydrogen peroxide. (**B**) Modulation of antioxidant related genes was observed in apigenin treatment. (**C**,**D**) Apigenin reversed the inhibition of enzyme activities of GPX and GR (^*^ *p* < 0.05, ^+^ *p* < 0.05 vs. control, ^#^ *p* < 0.05 vs. H_2_O_2_ treatment). Glutathione (GSH) is at the heart of one of the most important cellular antioxidant systems and capable of scavenging reactive oxygen species (ROS) and contributes to maintaining redox homoeostasis [26]. H_2_O_2_ significantly decreased the total GSH, which was further decreased by apigenin (Figure 5A). The biosynthesis of GSH is catalyzed by the action of two ATP-dependent enzymes, γ-glutamylcysteine synthetase (γ-GCS) and glutathione synthase (GS), and GCS catalyzes the formation of γ-glutamyl-cysteine from glutamate and cysteine in the presence of ATP, which is the rate-limiting step in biosynthesis [27]. Similarly oxidized dimer of GSH (GSSG) decreased by H_2_O_2_ was further inhibited by apigenin (Figure 5B). The decreased total GSH may have resulted from the downregulation of GCS transcription. In cells, GSSG can be regenerated by GR, and GR is responsible for maintaining the supply of reduced GSH [26]. The ratio of GSH/GSSG has been regarded as an index of oxidative stress [28]. However, apigenin alleviated the decreased ration of GSH to GSSH caused by H_2_O_2_ (Figure 5C), which was related to the increased mRNA expression of GR.

Apigenin is a flavone that exists widely in many fruits and vegetables such as onions, oranges, and tea [24]. Apigenin at 125 and 250 μg/mL significantly ameliorated the decreased cell viability caused by hydrogen peroxide (Figure 4A). In the hydrogen-peroxide-induced oxidative stress model in the MC3T3-E1 mouse osteoblastic cell line, pretreatment of cells with apigenin attenuated the reduced cell viability and upregulated the gene expression of SOD1, SOD2, and GPX [24]. However, apigenin reversed the increased mRNA expression of Nrf2 and GCS caused by H_2_O_2_ and decreased the mRNA expression of CAT but enhanced the transcription level of SOD (Figure 4B). SOD catalyzed the dismutation of O_2_^−^ to H_2_O_2_ [25]. Additionally, apigenin further increased the activity of GPX, which was slightly enhanced by H_2_O_2_ (Figure 4C). Different changes of GPX and CAT activity were observed in a similar cell model. GPX activity increased with higher H_2_O_2_ concentration, while CAT activity remained constant at different H_2_O_2_ treatments, which indicated that GPX was more active than CAT in scavenging H_2_O_2_ [21]. CAT played an important role as a primary defense enzyme against H_2_O_2_ at a low concentration but with a higher concentration of H_2_O_2_, GPX worked as a primary defense enzyme against oxidative damage [21]. Moreover, apigenin reversed the decreased activity of GR caused by H_2_O_2_ (Figure 4D). GPX and GR are two key enzymes involved in the glutathione (GSH) redox cycle, where GPX uses GSH to reduce organic peroxide and H_2_O_2_, and GR reduces glutathione disulfide (GSSG) to GSH in a nicotinamide adenine dinucleotide phosphate (NADPH)-dependent manner [25]. Since GPX and GR activity were increased by apigenin compared to the H_2_O_2_ treated group, intracellular GSH level was examined in the following study.

## 3. Materials and Methods

### 3.1. Material and Chemicals

*Plantago asiatica* L. was purchased from Tongrentang Chinese Medicine-Since 1669 (Beijing, China). Petroleum ether, dichloromethane, ethyl acetate, and methanol were analytically pure and obtained from Beijing Chemical Industry Group Co. LTD (Beijing, China). The 2,2-diphenyl-1-picrylhydrazyl (DPPH) was provided by Sigma-Aldrich LLC (St. Louis, Missouri, USA). Thin-layer chromatography GF254 and silica gel column chromatography (80–100 mesh, 100–200 mesh, and 200–300 mesh) were purchased from Qindao Haiyang Chemical Industry (Qingdao, China). Solution of 5% sulfuric acid ethanol was used as chromogenic agent. Aesculetin and apigenin (purity > 98%) were purchased from Aladdin Biochemical Technology Co. LTD (Shanghai, China).

### 3.2. Free Radical Scavenging Ability on DPPH and Absorbance Spectrum

DPPH scavenging activity was determined according to the method reported by Brand-Williams [29] with some modifications. A total of 25 mg DPPH was dissolved in 50 mL 80% ethanol as stock solution. DPPH stock solution was diluted with 80% ethanol at ratio of 1:2 (*v*/*v*) before measurement to prepare the work solution of DPPH. Each sample was diluted in a gradient ratio of 1:1 to 11 gradients. After dilution, 100 μL solution at all concentrations and 80% ethanol (as blank) was added into flat bottom 96-well plate. Then, the 100 μL DPPH work solution was added to each well, and the absorbance was determined at 517 nm after reaction at room temperature in the dark for 30 min. The eliminate percentage (E%) of DPPH at the steady state was determined using the following equation: E% = 1 − (Abs blank − Abs sample)/Abs blank. EC_50_, which is the concentration of substances (samples) discoloring 50% of the DPPH, was calculated by GraphPad Prism 5 (Version 7.04, Graphpad Software, San Diego, CA, USA, www.graphpad.com).

The absorbance of different extracts in the same concentration between 0 and 1000 nm wavelength was scanned by Microplate Reader (Spectra Max i3x, Molecular Devices, San Jose, CA, USA).

### 3.3. Cell Culture and H_2_O_2_ Exposure

Caco-2 cells were purchased from the Institute of Animal Science of CAAS (Beijing, China). Caco-2 cells were cultured in DMEM supplemented with 10% FBS, 1% penicillin, and streptomycin at 37 °C in humidified air containing 5% CO_2_.

Caco-2 cells (2 × 10^4^/well) grown for 24–48 h were allowed to attach to the culture plate before being pretreated with aesculetin or apigenin overnight. Then cells were treated with 1 mM H_2_O_2_ in DMEM without FBS for 2 h. The culture media and cells were collected for the further measurements.

### 3.4. Cell Viability and LDH Assay

Cell viability was measured using a CCK-8 kit in accordance with the manufacturer’s instructions (Dojindo, Kumamoto, Japan). Briefly, Caco-2 cells were seeded in a 96-well plate at 2 × 10^4^ cell per well and cultured overnight. Cells were pretreated with the same compounds isolated from *Plantago asiatica* L. for 18 h after plate attachment. Then, cells were then treated with H_2_O_2_ for 2 h. The culture media was collected for LDH (lactate dehydrogenase) assay and replaced by 200 μL DMEM supplemented with 10% CCK-8 per well and cultured at 37 °C for 2 h. Afterward, the absorbance was measured at 450 nm on a Microplate Reader (Spectra Max i3x, Molecular Devices, USA). The LDH activity in the cultural media was measured based on the reaction between LDH and lactic acid, which led to the generation of pyruvic acid, and pyruvic acid could become brown in alkaline environments with 2,4-dinitrophenylhydrazine via a commercial kit obtained from Nanjing Jiancheng Bioengineering Institute (Nanjing, China).

### 3.5. Measurement of Intercellular Transmembrane Resistance (TEER)

TEER was measured based on the method reported by Shao et al. [30]. Briefly, Caco-2 cells grown on a Transwell filter and transepithelial electrical resistance were monitored daily before differentiation by use of a Milicell Electrical Resistance System-2 (Millipore Corp., Bedford, MA, USA) and expressed as Ω × cm^2^.

### 3.6. Comet Assay

Comet assay, namely, single-cell gel electrophoresis assay, is a relatively convenient and sensitive technique for the analysis of DNA breakage in individual cells and commonly used for the investigation of antioxidants in intervention studies. The procedures of DNA strand breaks were determined by comet assay, according to the method reported by Fernández-Blanco with some modifications [31]. Briefly, Caco-2 cells (2.0 × 10^5^ cells/well) were seeded in 6-well plate and grew for 42 h. Then cells were treated with 0-1 mM H_2_O_2_ for 2 h followed by being suspended in prewarmed low-melting-point agarose. Additionally, suspension was rapidly transferred to precoated slide with agarose and covered with a coverslip. Coverslip was removed after gelling for 10 min at 4 °C, and a second low-melting-point agarose was added with gelatinization for 10 min at 4 °C. Then. slides were put into lysis buffer (2.5 M NaCl, 100 mM Na-EDTA, 10 mM Tris, 250 mM NaOH, 10% DMSO, and 1% Triton X-100) for 30 min at 4 °C and incubated in the fresh electrophoresis buffer (300 mM NaOH, 1 mM Na-EDTA) for 20 min to unwind the DNA after removing the residual lysate. After electrophoresis for 40 min (25 V, 300 mA), slides were washed with neutralization buffer (0.4 M Tris, pH 7.5) three times. Slides were stained with 500 μL PI (20 μg/mL), covered with a coverslip, and kept at 4 °C for 40 min. Slides were visualized under a fluorescence microscope (Lica universal microscope). At least 30 randomly selected single cells were analyzed by Comet Assay Software Project (http://casplab.com/). The DNA damage in cells was expressed as a percentage of total DNA content in the tail, tail moment, and olive tail moment (tail moment = tail length×tail DNA; tail moment = TailDNA% × (TailMeanX − HeadMeanX), equaling to ((percent of DNA in the tail) × (distance between the center of gravity of DNA in the tail and the of center of gravity of DNA in the head in x-direction)).

### 3.7. Transcription Levels Analysis by RT-PCR

Briefly, cells seeded in a 6-well plate after treatments were collected. Total RNA extraction was carried out by an Eastep Super Total RNA Extraction Kit (Peomaga Co., Shanghai, China). RNA quantity was measured by Nanodrop at 260 and 280 nm. Then, total RNA was reverse-transcribed into cDNA through a PrimeScripTMRT reagent Kit with gDNA Eraser (Perfect Real Time) (Takara, Japan), and gene expression was determined by SYBR Premix Ex TaqTM (Tli RNaseH Plus, Takara, Japan) in accordance with manufacturer’s protocol. Gene primers are reported in Table 2, and 2-ΔΔCt was calculated to express the gene expression level.

### 3.8. Measurements of Antioxidant Enzyme

Glutathione peroxidase (GPX) and glutathione reductase (GR) were analyzed using a commercial kit (Nanjing Jiancheng Bioengineering Institute, China). Briefly, the activity of GPX was determined based on the reaction between GSH and disulfide dinitrobenzoic acid (DNTB). Activity of GR was detected via changes of NADPH along with transformation of GSSG to GSH under catalysis of GR. Total GSH and reduced GSH were determined using a commercial GSH and GSSG kit (Beyotime biotechnology Co., Beijing, China). Briefly, DNTB and NADPH were converted to 5’-thionitrobenzoic acid (TNB) and NADP^+^ under catalysis by GR with GSSG and GSH. There was a positive correlation between the generation of TNB and total glutathione content.

### 3.9. Statistical Analysis

Statistical analysis was carried out using SPSS version 15 and data were expressed as mean ± SD. The differences between groups were analyzed with one-way ANOVA, and *p* < 0.05 was considered statistically significant.

## 4. Conclusions

The results of the present study have conclusively indicated that aesculetin and apigenin isolated from *Plantago asiatica* L. could ameliorate the Caco-2 cell damage caused by H_2_O_2_. Aesculetin protected cell from oxidative damage by activating Nrf-2 and its downstream genes such as SOD, CAT, and GCS and increasing the activity of GPX to enhance the intracellular antioxidant defense system. Apigenin exerted its protection against H_2_O_2_ mainly by improving the GSH antioxidant system, such as increasing the activity of GPX, GR, and the ration of GSH/GSSG. These findings suggest that aseculetin and apigenin may be bioactive substances for antioxidant function in *Plantago asiatica* L.

## Figures and Tables

**Figure 5:**
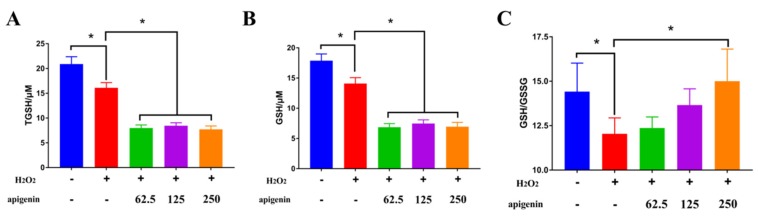
Modulation of apigenin on the total GSH (**A**), reduced GSH (**B**), and the ratio of GSH to GSSG (**C**) (^*^ *p* < 0.05).

**Table 1:** DPPH activity of different extracts of *Plantago asiatica* L. and its fractions.

Samples	EC_50_^1^ (mg/mL)	Samples from Fractions of E/M (10/1) Extract	EC_50_ (mg/mL)
Dichloromethane extract	0.351 ± 0.039^d^	F1	12.520 ± 2.106^f^
Ethyl acetate extract	0.160 ± 0.036^c^	F2	3.508 ± 0.145^e^
E/M (10/1) extract ^2^	0.117 ± 0.013^ab^	F3	0.499 ± 0.038^d^
E/M (5/1) extract	0.078 ± 0.012^a^	F4	0.114 ± 0.004^b^
E/M (2/1) extract	0.086 ± 0.016^a^	F5	0.230 ± 0.010^c^
Methanol extract	0.153 ± 0.021^c^	F6	0.064 ± 0.009^a^
*P*-value	<0.001	*P* value	<0.001

^1^ EC_50_: Concentration of substances (samples) discoloring 50% of the DPPH. ^2^ E/M (a/b): the ratio of ethyl acetate to methanol is a/b which was used for the extraction. Note: different superscripts in the same column differed significantly (*p* < 0.05).

**Table 2:** Primer for RT-PCR amplification.

Gene			Note
β-actin	Forward Primer	GGATGCAGAAGGAGATCACTG	NM_001498.4
	Revers Primer	CGATCCACACGGAGTACTTG	
Nrf2	Forward Primer	AAACCAGTGGATCTGCCAAC	NM_001101.5
	Revers Primer	GCAATGAAGACTGGGCTCTC	
SOD	Forward Primer	GTAATGGACCAGTGAAGGTGT	NM_000454.5
	Revers Primer	CAATTACACCACAAGCCAAACG	
CAT	Forward Primer	CGTGCTGAATGAGGAACAGA	NM_001752.4
	Revers Primer	AGTCAGGGTGGACCTCAGTG	
GCS	Forward Primer	GGCGATGAGGTGGAATACAT	M90656.1
	Revers Primer	CCTGGTGTCCCTTCAATCAT	
